# METTL16 Promotes Stability of SYNPO2L mRNA and leading to Cancer Cell Lung Metastasis by Secretion of COL10A1 and attract the Cancer-Associated Fibroblasts

**DOI:** 10.7150/ijbs.95375

**Published:** 2024-08-01

**Authors:** Jianlong Wu, Peng Ouyang, Rui Huang, Yao Cui, Zhihao Yang, Wei Xu, Rui Ma, Guoan Xiang, Wei Zeng, Wang Wu, Jian Li

**Affiliations:** 1 Department of General Surgery, Zhongshan City People's Hospital, Zhongshan, Guangdong 528403, P.R. China.; 2 Department of General Surgery, Affiliated Tumor Hospital of Zhengzhou University, Zhengzhou, Henan 450003, P.R. China.; 3 Department of Gastrointestinal surgery, The Affiliated Guangdong Second Provincial General Hospital of Jinan University, Guangzhou, Guangdong 510317, P.R. China.; 4 Department of General Surgery, The First Affiliated Hospital of Jinan University, Guangzhou, Guangdong 510632, P.R. China.; 5 Department of General Surgery, Yiyang Central Hospital Affiliated to Hunan University of Chinese Medicine, Yiyang, Hunan 413000, P.R. China.; 6 Department of Oncology, Henan Provincial People's Hospital, Zhengzhou, Henan 450003, P.R. China.; 7 Department of General Surgery, Henan Cancer Hospital, Zhengzhou, Henan 450003, P.R. China.; 8 Department of Biological Science, Mellon College of Science, Carnegie Mellon University. 5000 Forbes Avenue, Pittsburgh, PA, USA 15213.; 9 College of Bioengineering, Chongqing University, Chongqing 400044, P.R. China.; 10 Institute of Burn Research, Third Military Medical University, Chongqing 400038, P.R. China.

**Keywords:** Colorectal Cancer, N6-methyladenosine, Metastasis, Cancer-Associated Fibroblasts, Epithelial-Mesenchymal Transition

## Abstract

The occurrence of metastasis is a major factor contributing to poor prognosis in colorectal cancer. Different stages of the disease play a crucial role in distant metastasis. Furthermore, m6A has been demonstrated to play a significant role in regulating tumor metastasis. Therefore, we conducted an analysis of transcriptome data from high-stage and low-stage colorectal cancer patients in The Cancer Genome Atlas (TCGA) to identify genes associated with m6A-related regulation. We identified SYNPO2L as a core gene regulated by m6A, and it is correlated with adverse prognosis and metastasis in patients. Additionally, we demonstrated that the m6A writer gene Mettl16 can regulate the stability of SYNPO2L through interaction with YTHDC1. Subsequently, using Weighted Gene Co-expression Network Analysis (WGCNA), we discovered that SYNPO2L can regulate COL10A1, mediating the actions of Cancer-Associated Fibroblasts. SYNPO2L promotes the secretion of COL10A1 and the infiltration of tumor-associated fibroblasts, thereby facilitating Epithelial-Mesenchymal Transition (EMT) in tumor cells and making them more prone to distant metastasis.

## Introduction

N6-Methyladenosine (m6A) is a common modification found in RNA 1 that affects the stability, translation, and conformation of transcripts, thereby influencing cellular activities 2. The function of m6A is determined by its writers, erasers, and readers 3. The process of m6A is an important study that worth in-depth research. For example, m6A methylation can promote the degradation and turnover of mRNA, while enhancing the efficiency of translation. Given mRNA's essential role in tumor, the importance of m6A is underscored by its ability to affect the expression levels of certain key oncogenes or suppressor genes 4. Recently, another m6A methyltransferase, METTL16, has been identified, though the number of its targets seems to be more limited 5. Like METTL3, METTL16 contains a Rossmann fold, which is typical in methyltransferases, and uses S-Adenosyl methionine (SAM) as a methyl donor. However, it appears to have additional regulatory domains and RNA-binding domains 6. Interestingly, in global study of identifying mRNA-binding proteins, polyadenylated mRNA was isolated along with endogenous proteins, and METTL16 was frequently captured, while the components of the METTL3/14 complex were not 6. This suggests that METTL16 may be a very important m6A regulatory target, which deserves our research. Our studies have found that METTL16 plays an important role in regulating the progression of colorectal cancer.

However, as a target affecting mRNA stability, METTL16 is likely to exert its effects through some specific targets. Our research has first identified SYNPO2L as a potential important target of METTL16, which may influence the progression and metastasis of tumors. Intriguingly, there is little research on the role of SYNPO2L in regulating tumors. In fact, the discovery of this gene was not very early. Beqqali first studied the function of the product of SYNPO2L gene, and named it Cytoskeleton Heart enrichment Actin associated protein (CHAP) 7. Their research suggests that SYNPO2L may be involved in the development of cardiomyocytes, indicating that this gene may play an important role in fibroblasts or cardiac cells. Our studies also find that SYNPO2L has a significant role in affecting the chemotaxis of tumor fibroblasts. Furthermore, we discovered that this gene is associated with poor prognosis in patients with colorectal tumors. However, how SYNPO2L attracts and influences fibroblasts is an urgent problem to be addressed. We found that SYNPO2L can affect the secretion of COL10A1 in tumor cells, thereby mediating the involvement of tumor-associated fibroblasts. Type X collagen α1 (COL10A1) is part of the collagen family 8. The COL10A1 gene encodes the α chain that forms type X collagen, this procollagen exists in the form of proliferating chondrocytes during endochondral ossification 8. Studies have shown that COL10A1 can promote tumor fibroblast enrichment, thereby facilitating tumor metastasis 9. Our research has also found that COL10A1 can chemotactically enrich tumor fibroblasts.

## Material and Methods

### Bioinformatics Analysis

The gene expression information and clinical factors of colorectal cancer came from The Cancer Genome Atlas (TCGA) database. Differential gene expression analysis was carried out using colorectal cancer RNA-seq data from TCGA to identify differences in expression between metastatic and non-metastatic tumor tissues. Gene differential analysis used R's limma package. Patient prognosis differences were analyzed using R's survival analysis package. Functional enrichment analysis was based on the metscape platform, including Molecular Signatures Database (MSigDB) hallmarks and Kyoto Encyclopedia of Genes and Genomes (KEGG) 9. The background genes for enrichment analysis used all genes in the genome. Items with a P value <0.05, containing at least three genes and an enrichment factor >1.5 were collected and grouped based on the similarity of their members. P values were calculated based on the cumulative hypergeometric distribution, and q values were calculated according to the Benjamini-Hochberg (BH) method^10^. Correlation networks were drawn through R's correlation network package based on the correlation between proteins^11^. Forest plots were drawn using R's correlation analysis package. Heatmaps of differential genes were drawn using R's heatmap package, and gene differences were contrasted through the colors of the heatmap. For the survival analysis of tumor patients, we analyzed TCGA's survival data through gepia (http://gepia.cancer-pku.cn/detail.php?gene). To validate survival prognosis, we also analyzed survival prognosis information from public databases through kmplot (https://kmplot.com/analysis/index.php?p=service). After selecting samples, we extracted the expression matrix from the samples and then used the “estimate” R package to calculate the immune purity of the expression matrix. We performed the ssGSEA method on each sample and calculated the total matrix content (Cancer associated fibroblastsScore) and combination (EstimateScore) through the ESTIMATE algorithm^12^.

### Establishment of Weighted Correlation Network Analysis

Using the WGCNA package in r3.6.2, we studied the co-expression relationships based on gene expression^13^. Because genes with low expression or no variation generally represent noise, we performed variance filtering on genes, selecting the top 20% of expressed genes for subsequent analysis. First, the “good Samples Genes” function in the WGCNA package was used to evaluate the input genes and samples. To construct a weighted gene network, it is necessary to choose a soft thresholding power β for co-expression to enhance similarity and calculate adjacency. The β value is selected based on the “pick Soft Threshold” function, which performs network topology analysis and selects an appropriate soft threshold based on the criteria for an approximate scale-free topology. The adjacency matrix is transformed into a Topological Overlap Matrix (TOM). TOM measures a gene's network connectivity, defined as the sum of its adjacency to all other genes, and is used for network generation. In order to classify genes with similar expression patterns into gene modules, average linkage hierarchical clustering is performed based on TOM dissimilarity measures. Correlation analysis of gene expression in the module with external clinical features and intra-module analysis are conducted to determine whether gene expression is consistent with clinical relevance.

### Patient and Tissue Samples

35 cases of formalin-fixed paraffin-embedded (FFPE) colorectal cancer specimens with pulmonary metastasis and their matched adjacent non-tumor tissues were selected from patients enrolled between 2009 and 2022 at the First Affiliated Hospital of Jinan University and the Second People's Hospital of Guangdong Province. None of the patients underwent chemotherapy or radiotherapy before surgery. These studies were conducted in accordance with the International Ethical Guidelines for Biomedical Research Involving Human Subjects. The studies were carried out after approval by the Institutional Review Boards. Written consent was obtained from the subjects before the start of the study. From June 2019 to December 2022, fresh tissue from primary colorectal cancer patients and matched normal tissues were collected from the First Affiliated Hospital of Jinan University for mRNA analysis. All samples were pathologically confirmed, and tumors were graded according to the World Health Organization standards and the TNM staging system of the American Joint Committee on Cancer. Follow-up information and specific clinical pathology data were available for all patients. Informed consent was obtained from the patients participating in the study. All experiments were conducted in accordance with the Declaration of Helsinki and approved by the ethics committees of the First Affiliated Hospital of Jinan University and the Second People's Hospital of Guangdong Province.

### Cells, Cell Culture, and Co-culture

Human colorectal cancer cell lines (SW480, HCT116) and mouse-derived colorectal cancer cell line (MC38), as well as human normal fibroblast cell line, were obtained from the Cell Bank Bank, Chinese Academy of Sciences (Shanghai, China). All cell lines were mycoplasma-free and identified through Short Tandem Repeat (STR) DNA analysis. Cells were incubated in DMEM or RPMI 1640 medium (Gibco, USA) with 10% fetal bovine serum (FBS; Gibco, USA) and 1% penicillin-streptomycin, in a humidified atmosphere of 5% CO2/95% air at 37°C. For co-culture experiments, human fibroblast cell line (Shanghai Fusheng Industrial Co., Ltd.) (5×10^5) were placed in the lower chamber of a 12-well plate, and colorectal cancer cells (5×10^5) were added to the upper chamber in 0.4 mm diameter Transwell inserts (Millipore).

For knockdown of SYNPO2L, METTL16, YTHDC1, and COL10A1, lentiviral vectors containing shRNA for SYNPO2L, Mettl16, YTHDC1, and COL10A1 and negative controls were fused and cloned into the pLKO.1 vector. For overexpression of SYNPO2L, Mettl16, YTHDC1, and COL10A1, lentiviral vectors for overexpressing SYNPO2L, Mettl16, YTHDC1, and COL10A1 and negative controls were fused and cloned into the plv vector. Plasmids were transfected into cells using Lipofectamine iMAX (Invitrogen, USA) according to the manufacturer's protocol. The shRNA sequences are as follows: Mettl16 sh1: GGTATTTCCTCGCAACAGAAGTGGATGATATGTGTTTCAAC. METTL16 sh2: TTACTTGGAGCAACCTTGAATGGCTGGTATTTCCTCGCAAC. YTHDC1 sh1: CCCTGAGTTTCACCAGAGACCAGGGTATTTAAAGGATCCAC. YTHDC1 sh2: TATCAAGTAGTGCCTCCAGAGAACCTTATAAGAATCAACCT. Human SYNPO2L sh1: GGGCAGCGGCCAACTACCACCTCGGTTATTTTCCGGCCTTT. Human SYNPO2L sh2: ATTTTATGCGGCATCAGCCCTATCAACTTAAAACTGCCATG. Human COL10A1 sh1: TTCTAGGAAACATCCAGGAGGTATCATATAACTTTGTAGAA. Human COL10A1 sh2: GTTCTTCATTCCCTACACCATAAAGAGTAAAGGTATAGCAG. Mouse SYNPO2L sh1: GAGCCCCCATCCCTGCACCAAGTATCTTTAACAGGTCAGCG. Mouse SYNPO2L sh2: AGAGAGGAGTGCCTGTAGCATGTAGAGATATGTGCACAGCA. Mouse COL10A1 sh1: GAACGGCACGCCTACGATGTACACGTATGATGAGTACAGCA. Mouse COL10A1 sh2: CTAGCCCCAAGACACA.

### Quantitative Reverse Transcription PCR

Total RNA was extracted from tumor and normal tissues using TRIzol reagent (Ambion, Austin, TX, USA). After reverse transcription using cDNA as a template, mRNA levels were detected using TB Green®Premix Ex Taq™II (Invitrogen) in an ABI PRISM 7500 system (Applied Biosystems, Foster City, CA, USA). RNA expression levels were normalized to glyceraldehyde-3-phosphate dehydrogenase (GAPDH). mRNA expression levels were normalized to GAPDH.

### RNA Stability Measurement

To measure the RNA stability of DEPTOR, cells were treated with 4µg/mL Actinomycin D at 0, 3, and 6 hours, respectively. Then total RNA was extracted using TRIzol reagent before qRT-PCR. At each treatment time, cell mRNA expression levels were measured and normalized to β-actin.

### MeRIP-qPCR Assay

m6A Immunoprecipitation (MeRIP) was performed using the Magna MeRIP™ m6A Kit (#17-10499, Merck Millipore, MA) based on the manufacturer's instructions. Briefly, purified mRNA was digested with DNase I and then fragmented into ~100nt fragments using RNA fragmentation reagents, incubated at 94°C. After fragmentation, termination buffer was added, followed by standard ethanol precipitation and collection. 12μg of anti-m6A antibody was pre-incubated with 50μl beads in IP buffer (150mM NaCl, 0.1% NP-40, 10mM Tris-HCl, pH 7.4) at room temperature for 1 hour. Next, 6μg of fragmented mRNA was added to the antibody-bead mixture and incubated at 4°C on a rotator for 4 hours. After thorough washing, the immunoprecipitated mixture was digested with high-concentration proteinase K, and the bound RNA was extracted using phenol-chloroform method and ethanol precipitation for qPCR analysis or library construction. qPCR analysis identified m6A modifications in the analysis based on precise primers.

### RNA Immunoprecipitation (RIP)

RIP experiments were conducted as previously described^2^. Briefly, cell lysates were incubated with 1 µg of antibodies specific for IgG, m6A overnight at 4 °C, then 20 µl of washed magnetic beads were added to each reaction and incubated for 2 hours at 4 °C. After three washes, the target RNA in the purified immunoprecipitated complex was used for further qRT-PCR analysis. The relative enrichment of RNA was normalized to input.

### Luciferase Reporter Assay

cDNA containing the full-length sequence of Synpo2l with a firefly luciferase reporter was cloned into the pGL3 control vector (Promega). For mutant 1 and 2 reporter plasmids, adenosine G and C in the marked m6A motif were replaced with A. Pre-treated cancer cells were seeded in 6-well plates, then 0.5μg of wild-type or mutant Synpo2l reporter plasmid and 25ng pRL-TK plasmid (Renilla luciferase reporter vector) were co-transfected using the jetPRIME Polyplus kit. After 24-36 hours, cells were collected and luciferase activity was assessed using the Dual-Glo Luciferase System (Promega), normalized to pRL-TK activity. Each experiment was performed in triplicate.

### EDU

The cell suspension should be added to each well of a 96-well plate to achieve a cell count of 1*10^4 cells per well. The plate should then be incubated overnight in an incubator. After incubating each well with 10μM EDU (Beyotime, Shanghai, China) for 2 hours, the culture medium should be removed and 1ml of fixative (Beyotime, Shanghai, China) should be added. The fixative should be left at room temperature for 15 minutes before being removed. The cells should then be washed three times with 1ml of washing solution per well. After removing the washing solution, 1ml of permeabilization solution should be added to each well and incubated at room temperature for 10-15 minutes. Next, the Click reaction solution (Beyotime, Shanghai, China) should be added, and the plate should be incubated at room temperature in the dark for 30 minutes. Finally, Hoechst 33342 (Beyotime, Shanghai, China) can be used for nuclear staining. The cells can then be counted and photographed using fluorescence inversion microscopy.

### Transwell Migration and Invasion Assay

Migration and invasion assays were performed using Transwell chambers (Millipore, Billerica, USA). Transfected cells were seeded in the upper chamber with serum-free medium (2.5×10^4 cells), while the bottom was seeded with DMEM containing 10% fetal bovine serum. For invasion assays, the culture plates were coated with Matrigel (BD Biosciences, Franklin Lakes, NJ, USA), and subsequent steps were similar to the migration assay. Cells were fixed and stained with crystal violet after 24 hours of migration or invasion. Migrated and infiltrated cells were counted under an inverted microscope. The number of migrating or invading cells was quantified by counting the number of cells in 10 random fields at a ×100 magnification.

### Western Blot Analysis

Total protein was extracted from HCC cells, then 20 μg of protein was separated using 10% SDS-PAGE and transferred to a PVDF membrane (Bio-Rad Laboratories, Hercules, CA, USA). The membranes were probed overnight with respective primary antibodies (Table S6). Subsequently, the membranes were incubated with enzyme-labeled goat anti-mouse or anti-rabbit IgG antibodies (zsbb - bio, China). Protein bands were visualized using an enhanced chemiluminescence kit (Amersham, Little Chalfont, UK). The experimental details are as previously described^14^.

### Immunohistochemistry (IHC)

Immunohistochemical processing was performed on formalin-fixed paraffin-embedded sections. Streptavidin-Biotin Complex Immunoperoxidase (SP-IHC) was used for the immunohistochemistry of Mettl16, E-cadherin, SYNPO2L, Vimentin, Mesenchymal Cancer associated fibroblasts Cell Marker (Mesenchymal Cancer associated fibroblasts Cell Marker (CD44, CD45, CD90 CD29, CD105) Antibody Panel - Human (ab93758)), and COL10A1 primary antibodies. Immunohistochemical methods were carried out as previously reported^14^. Staining intensity was classified into four grades: 0, none; 1, weak; 2, moderate; 3, strong. The percentage of tumor cell-specific positive staining was categorized into the following levels: 0 (<5%), 1 (6%-25%), 2 (26%-50%), 3 (51%-75%), and 4 (>75%). The final score was represented by multiplying the staining intensity with the percentage of specific positive staining tumor cells.

### Plate Colony Formation Assay

Cells were prepared in suspension using routine digestion and passaging methods for exponential cell growth. Cell suspensions were mixed repeatedly to ensure full dispersion of cells, with a single-cell percentage greater than 95%. Cells were counted, the concentration was adjusted with medium, and the cells were plated. Cell suspensions were diluted in multiple ratios according to cell proliferation capability. 5 ml of cell suspension was inoculated into a six-well plate (60 mm diameter) at densities of 50, 100, and 200 cells per well, gently shaking the culture dish to evenly disperse the cells. The culture plates were incubated under conditions of 37°C and 5% CO2 for 2-3 weeks, with fresh medium replaced timely according to changes in medium pH. Once visible clones appeared in the culture dish, the culture was terminated, and the medium was discarded. PBS solution was carefully added twice, and cells were air-dried. Cells were fixed with methanol for 15 minutes and air-dried after discarding methanol. Cells were stained with Giemsa solution for 10 minutes. The solution was gently washed off with running water, and cells were air-dried.

### Animal Experiments

Five-week-old male BALB/c athymic nude mice were purchased from the Animal Center of the First Affiliated Hospital of Jinan University and randomly divided into experimental and control groups. 2 × 10^6 cells stably overexpressing or silencing SYNPO2L, Mettl16 in SW480 cells were injected into the right abdomen of the mice to observe tumor growth. Tumor volume was monitored weekly after injection and calculated using the formula 0.5 × a^2 × b (where a and b represent the short and long tumor diameters, respectively). Four weeks later, mice were killed, tumors were removed and weighed for histological analysis and further study. For tail vein injection in mice, we injected 2 × 10^6 cells stably overexpressing or silencing SYNPO2L or COL10A1 in mouse cell lines mc38 and sw480 via tail vein and monitored tumor growth and mouse vitals. As mice contain mouse fibroblasts, injecting mc38 allows for the study of the effect of fibroblasts on tumors. However, as mice do not contain human fibroblasts, injecting sw480 simulates the state of experiments without human fibroblasts. This study was authorized by the Ethics Committee of the First Affiliated Hospital of Jinan University.

### CCK8

Harvested cells were seeded into a 96-well plate at a density of 1x10^4 cells per well, with a total volume of 200µl of culture medium. After a specified period of cell treatment, Cell Counting Kit-8 (CCK-8) from MedChem Express, China (20µl per well) was added. The cells were then incubated at 37°C for 3 hours. Subsequently, the plate absorbance was measured using an automated microplate spectrophotometer from Bio-Rad Laboratories, Inc., Hercules, CA.

### Statistical Analysis

All data were analyzed using GraphPad Prism software (GraphPad, USA) and presented as mean ± standard deviation. Differences between two or more groups were assessed using Student's t-test, one-way ANOVA, or two-way ANOVA. Paired t-test was used to compare mRNA expression between paired tumor tissues. Survival curves were analyzed by Kaplan-Meier method and log-rank test. P values < 0.05 were considered as statistically significant.

## Results

### Exploring Core Genes in Regulating Distant Metastasis through m6A Using Bioinformatics in Public Databases

**Figure 1:**
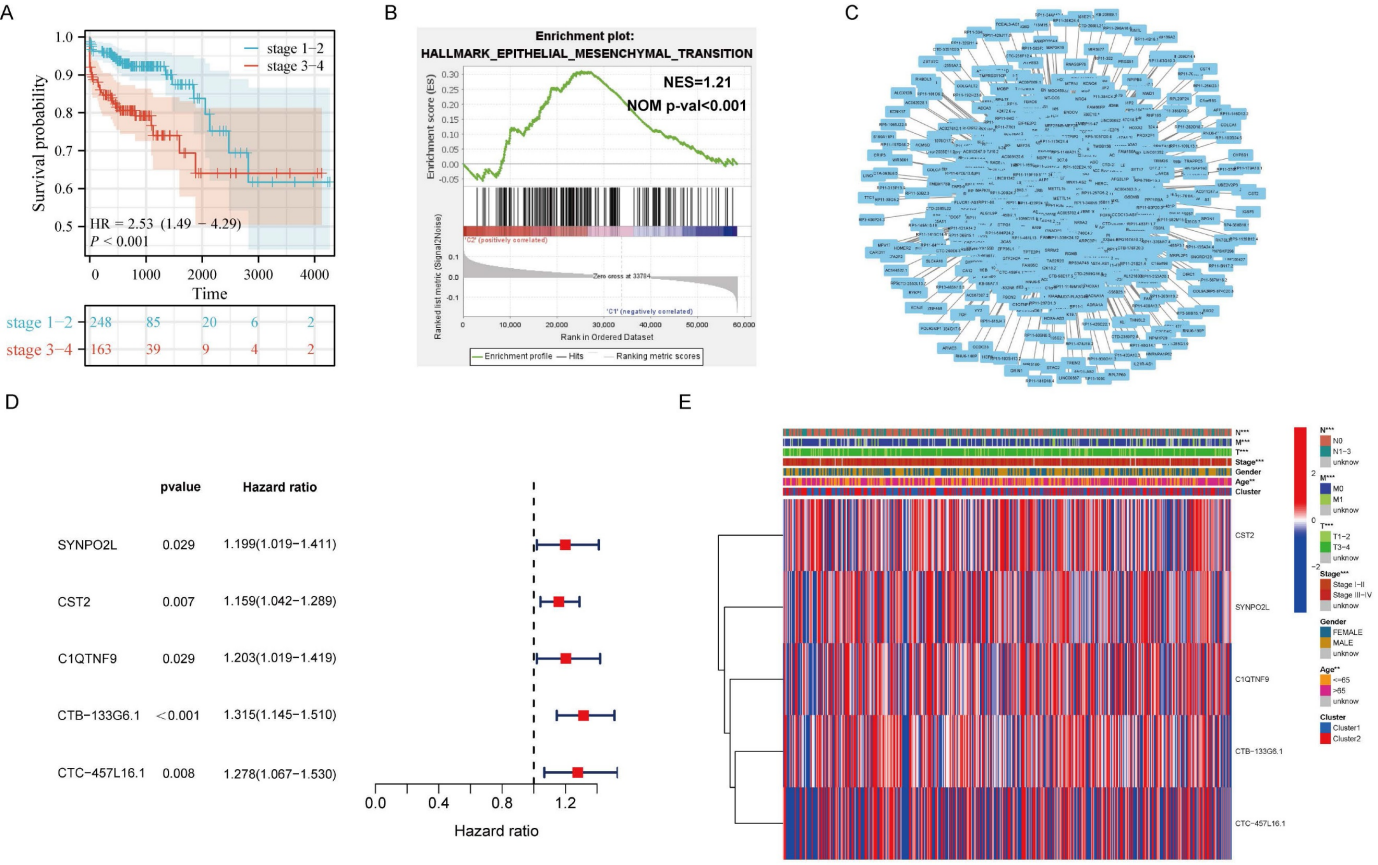
** Analysis of Transcriptome Data from TCGA Colorectal Cancer Patients Reveals m6A-regulated Core Gene SYNPO2L as a Significant Factor in Patient Staging.** (A) KM survival analysis; (B) Hallmark GSEA enrichment analysis, NES= 1.21, NOM p-val < 0.001; (C) Protein interaction network map for m6A-related genes and all associated genes; (D) Survival analysis forest plot for significant differences between m6A related genes and all associated genes; (E) Heatmap analysis of genes with significant differences between m6A-related genes and all associated genes in relation to TNM, gender, age, staging, etc. *P < 0.05, **P < 0.01, ***P < 0.001, ****P < 0.0001.

It is well-known that m6A plays a significant role in regulating the occurrence and development of tumors. However, it's still unclear which genes that m6A influences in tumor progression and metastasis. Therefore, we included 451 colorectal cancer patients from the TCGA colorectal cancer database. Through corresponding analysis of patient metastasis and survival prognosis, we first confirmed that patients with late-stage colorectal cancer have significantly worse survival prognosis compared to those with early-stage cancer, showing a noticeable statistical difference (Figure 1A). Further enrichment analysis found that EMT is the main cause leading to late stages in patients (Figure 1B). Next, we analyzed all genes related to m6A expression. Through correlation analysis and protein interaction mapping, we identified 1214 genes associated with m6A expression (Figure 1C). To filter out noise and select core genes truly related to patient prognosis, we conducted survival prognosis analysis on these 1214 core genes. We found that SYNPO2L, CST2, C1QTNF9, CTB-133G6.1, and CTC-457L16.1 are related to m6A expression and patient survival prognosis (Figure 1D). We then compared these five genes through a heatmap, focusing on their differences between late and early stage patients. We observed that the distant metastasis, lymph node metastasis, invasion depth and ages of late stage patients, and the expression of SYNPO2L are significantly related to late stage cancer in patients (Figure 1E).

### m6A Writer Gene METTL16 Positively Correlates with SYNPO2L Expression

**Figure 2:**
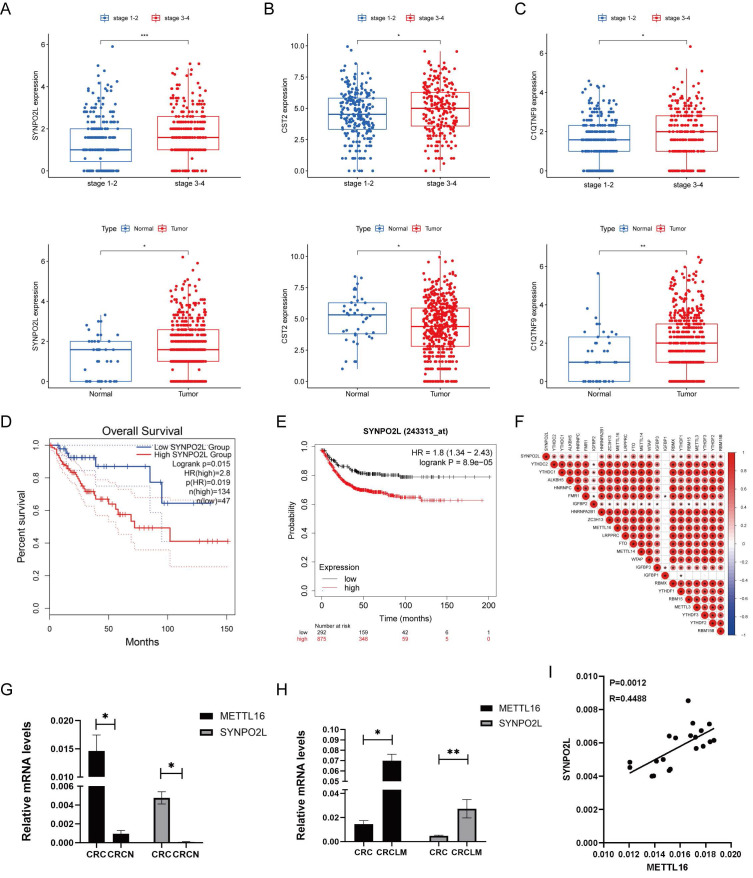
** METTL16 Expression Significantly Correlates with The Expression of SYNPO2L, A Core Gene Related to Patient Survival Prognosis.** (A) Statistical analysis of SYNPO2L mRNA expression in cancer and adjacent cancer tissue, and in late and early stage patients; (B) Statistical analysis of CST2 mRNA expression in cancer and adjacent non-tumor tissues, as well as in patients with high and low stage cancer; (C) Statistical analysis of C1QTNF9 mRNA expression in cancer and adjacent non-tumor tissues, as well as in patients with high and low stage cancer; (D) Overall survival analysis based on SYNPO2L expression levels in colorectal cancer patients from the TCGA database; (E) Overall survival analysis based on SYNPO2L expression levels in colorectal cancer patients from the TCGA database; (F) Correlation analysis of SYNPO2L with m6A-related genes; (G) qPCR analysis of METTL16 and SYNPO2L expression in CRC cancer and adjacent non-tumor tissues; (H) qPCR analysis of METTL16 and SYNPO2L expression in CRC cancer and lung metastasis; (I) qPCR analysis of the correlation between METTL16 and SYNPO2L expression. *P < 0.05, **P < 0.01, ***P < 0.001, ****P < 0.0001.

Through our bioinformatics analysis, examining genes associated with m6A gene expression, we found a positive correlation between SYNPO2L and m6A gene expression. SYNPO2L also shows a significant correlation with the late stages of patients (Figure S1A). Further analysis revealed that SYNPO2L expression is significantly higher in patients with late stages compared to early stages, and its expression in tumors is much higher than in normal tissues (Figure 2A). The differences in CST2 and C1QTNF9 are not as pronounced as in SYNPO2L (Figures 2B, C). Comparing survival prognosis of patients in TCGA and GEO databases, we found that SYNPO2L is significantly associated with poor prognosis in both databases (Figures 2D, E). Our previous research discovered that m6A plays a crucial role in regulating SYNPO2L. Consequently, through correlation analysis, we found that METTL16 has the strongest correlation with SYNPO2L expression (Figure 2F). Also, using qPCR on RNA from our colorectal cancer patient database, we found that these two genes are significantly overexpressed in tumors compared to normal tissues (Figures 2G, H), highlighting their importance in tumor regulation. To further verify whether METTL16 is related to SYNPO2L mRNA expression, we conducted correlation analysis on our qPCR results and found a close correlation between METTL16 expression and SYNPO2L (Figure 2I).

### SYNPO2L is a Key Gene in Tumor Progression

**Figure 3:**
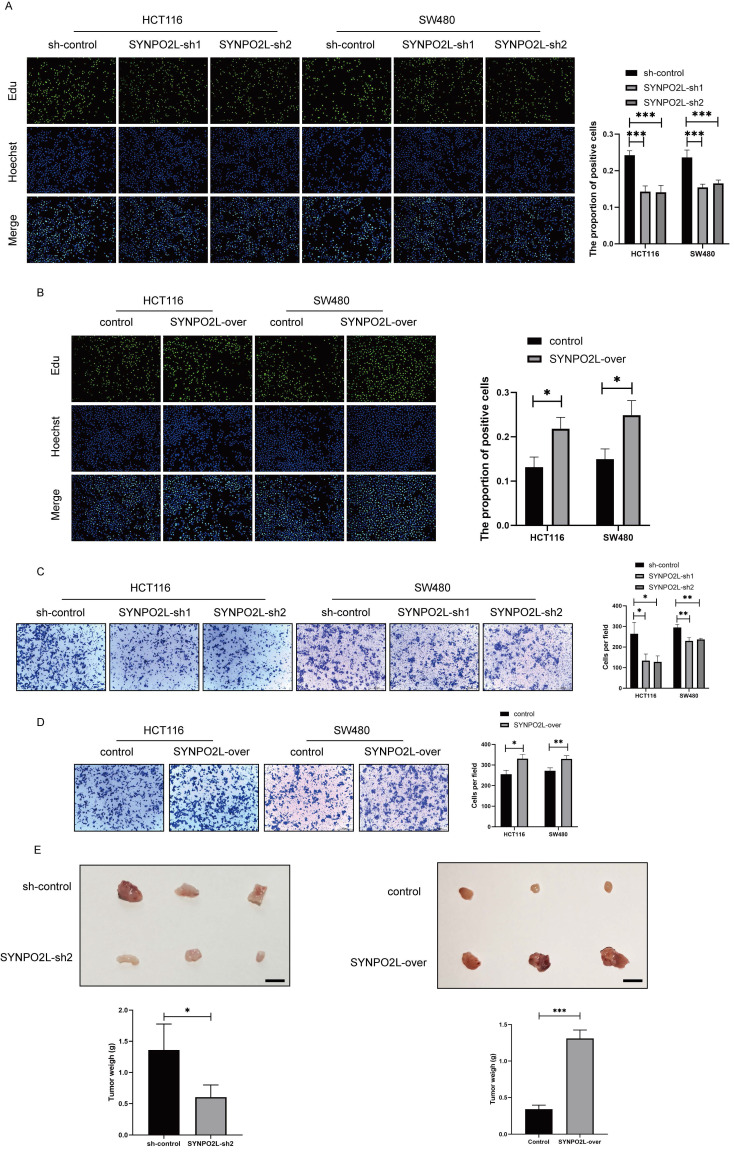
** SYNPO2L Significantly Impacts Tumor Cell Proliferation and Migration.** (A-B) EDU experiment and statistical bar graph after SYNPO2L knockdown or overexpression; (C-D) Transwell migration experiment and statistical bar graph after SYNPO2L knockdown or overexpression; (E) Subcutaneous tumor formation experiment in nude mice, comparing tumor size and weight after injecting CRC cell lines transfected with Sh-Control/SYNPO2L-Sh2/control/SYNPO2L-over, and a statistical bar graph comparing the weight of the xenograft tumors. *P < 0.05, **P < 0.01, ***P < 0.001, ****P < 0.0001. The length of the ruler is 1cm.

Our prior research indicates that SYNPO2L is an important factor affecting tumor stages and poor patient prognosis. We first validated its impact on tumor cells by knocking down SYNPO2L in vitro in colorectal cancer cell lines (Figure S1B, C). Through colony formation experiments, we found that knocking down SYNPO2L significantly slowed down cell proliferation and migration, indicating its crucial role in tumor growth (Figure 3A-D). Furthermore, through in vivo subcutaneous tumorigenesis experiments in nude mice, we injected the SYNPO2L-knockdown cell line subcutaneously into nude mice and observed tumor growth in the mice. We found that knocking down SYNPO2L significantly affects tumor growth (Figure 3E). Both in vivo and in vitro experiments suggest that the absence of SYNPO2L inhibits tumor growth. We then investigated whether supplementing sufficient SYNPO2L would affect tumors. By constructing an overexpression lentivirus plasmid of SYNPO2L and introducing it into colorectal cancer cell lines in colony formation experiments, we observed that overexpression of SYNPO2L significantly promoted tumor cell growth (Figure 3E). Similar results were observed in in vivo subcutaneous tumorigenesis experiments with nude mice, where overexpression of SYNPO2L effectively promoted tumor growth (Figure 3E).

### METTL16 Inhibits Tumors by Affecting SYNPO2L Expression

**Figure 4:**
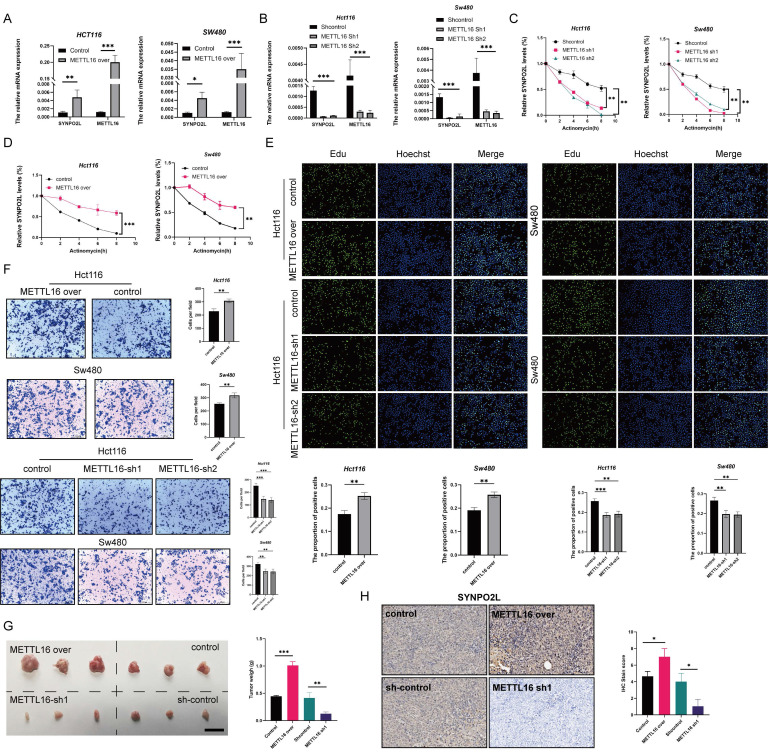
** METTL16 Promotes Tumor Proliferation and Migration by Enhancing SYNPO2L mRNA Stability Through m6A Modification.** (A-B) qPCR analysis of METTL16 and SYNPO2L expression in colorectal cancer cell lines with overexpression/knockdown of METTL16; (C-D) Analysis of SYNPO2L mRNA degradation rate after METTL16 knockdown and Actinomycin D treatment; (E) EDU experiment and statistical bar graph after METTL16 knockdown or overexpression; (F) Transwell migration experiment and statistical bar graph after METTL16 knockdown or overexpression; (G) Subcutaneous tumorigenesis experiment in nude mice, comparing the size of xenograft tumors in nude mice after injection of CRC cell lines stably transfected with METTL16-Sh1/METTL16-over/control, along with a statistical bar graph comparing the weight of the xenograft tumors; (H) Immunohistochemical analysis of xenograft tumors in nude mice to detect METTL16 protein expression, and a comparative statistical bar graph of immunohistochemical scoring. *P < 0.05, **P < 0.01, ***P < 0.001, ****P < 0.0001. The length of the ruler is 1cm.

In fact, SYNPO2L plays an obvious role in promoting tumor growth. However, our previous studies revealed that METTL16 is a key factor influencing SYNPO2L, especially the role of METTL16 on promoting SYNPO2L. Thus, it is crucial to study the effect of METTL16 on SYNPO2L. We first examined whether METTL16 could affect the expression of SYNPO2L. We first overexpressed and knocked down METTL16 through lentivirus and then did qPCR. We found that overexpression of METTL16 significantly enhanced SYNPO2L expression (Figure 4A). When METTL16 was knocked down, SYNPO2L expression significantly decreased (Figure 4B). Since METTL16 is not a classic m6A writer, it was necessary to verify whether METTL16 influences SYNPO2L through m6A methylation. We used Actinomycin to induce mRNA degradation by interfere with mRNA transcription to test whether METTL16 could affect the stability of SYNPO2L. Through the knockdown of METTL16 and the use of Actinomycin, we found that knocking down METTL16 significantly reduced the stability of SYNPO2L (Figure 4C). Conversely, overexpression of METTL16 decreased the degradation of SYNPO2L (Figure 4D), suggesting that METTL16 affects SYNPO2L mRNA stability.

However, to confirm METTL16 functions through SYNPO2L, we need more evidence. Our previous research showed that SYNPO2L affects tumor growth. We then examined whether METTL16 affects tumor growth. Overexpressing METTL16, we observed significantly more cell growth and migration compared to the control group (Figure 4E, F, Figure S2A-C). Moreover, knockdown of METTL16 significantly inhibited tumor growth and migration (Figure 4E, F, Figure S2A-C). In vivo experiments also validated the role of METTL16 (Figure 4G). Interestingly, when METTL16 was knocked down while SYNPO2L was overexpressed, tumor growth and migration were similar to the control group (Figure S2 A-C). Conversely, overexpressing METTL16 while knocking down SYNPO2L negated the effects of METTL16 (Figure S2A-C). Double knockdown of both METTL16 and SYNPO2L almost halted cell growth and migration (Figure S2A-C), while their simultaneous overexpression significantly enhanced their effects (Figure S2A-C). This could be explained as SYNPO2L RNA levels increase with overexpression, and METTL16 stabilizes SYNPO2L expression, enhancing translation. These experiments further illustrate that METTL16 inhibits tumors primarily through SYNPO2L. In vivo experiments with METTL16 knockdown in mice further confirmed that knocking down METTL16 indeed inhibits SYNPO2L expression (Figure 4H).

### YTHDC1 Specifically Recognizes SYNPO2L, While METTL16 Promotes the Stability of SYNPO2L mRNA through m6A Methylation

**Figure 5:**
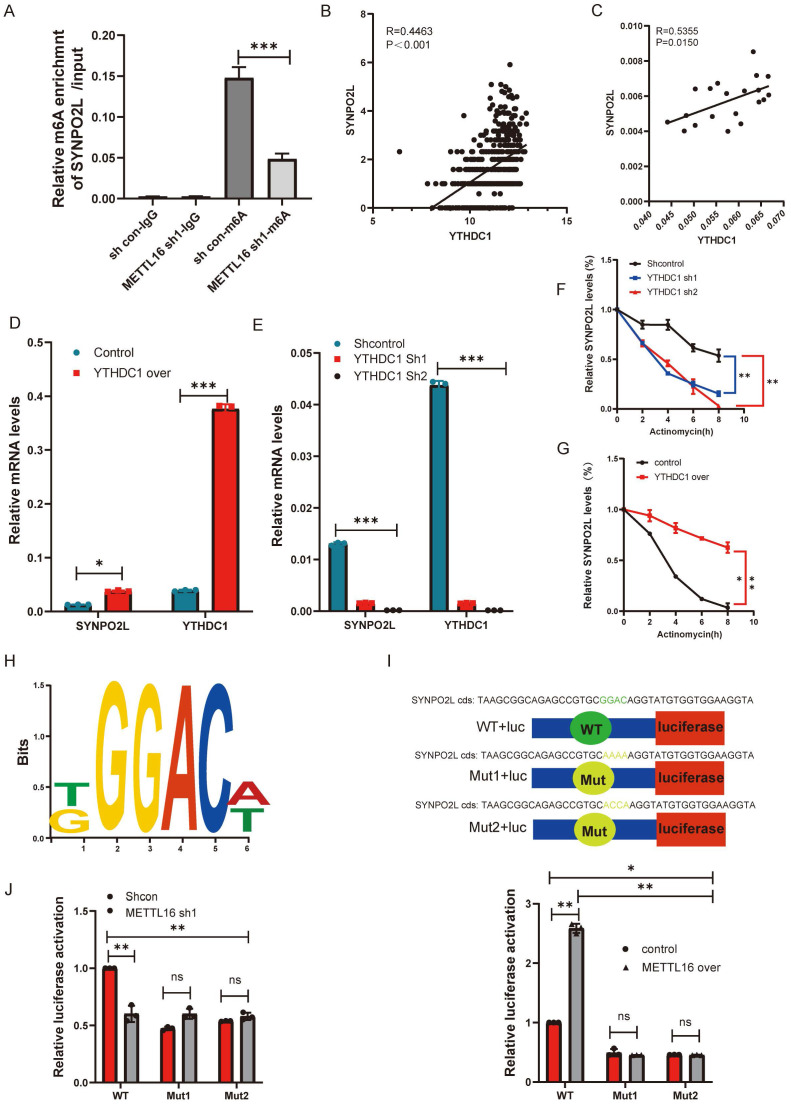
** YTHDC1 Recognizes and Promotes SYNPO2L mRNA Stability Through METTL16-Mediated m6A Modification.** (A) MeRIP-qPCR assessment of SYNPO2L m6A levels and statistical bar graph analysis after METTL16 knockdown;(B) TCGA database analysis of the correlation between YTHDC1 and SYNPO2L mRNA expression; (C) qPCR analysis of the correlation between YTHDC1 and SYNPO2L expression; (D-E) qPCR analysis of YTHDC1 and SYNPO2L expression in colorectal cancer cell lines with overexpression/knockdown of YTHDC1; (F-G) Analysis of SYNPO2L mRNA degradation rate after YTHDC1 knockdown/overexpression and Actinomycin D treatment; (H) Diagram of SYNPO2L gene stability regions; (I-J) Luciferase reporter gene analysis after mutating SYNPO2L gene stability regions and statistical bar graph. *P < 0.05, **P < 0.01, ***P < 0.001, ****P < 0.0001.

From previous experiments, the relationship between METTL16 and SYNPO2L is quite clear. METTL16, as an m6A methylation writer, targets SYNPO2L, thereby enhancing its mRNA stability. To prove this, we knocked down Mettl16 and performed MeRIP-qPCR, observing a significant decrease in SYNPO2L (Figure 5A), indicating that METTL16 mediates m6A methylation on SYNPO2L.

Although the writers and readers of SYNPO2L are quite apparent, we wonder which specific reader is involved. From correlation analysis, we found that YTHDC1 has the highest correlation with SYNPO2L expression among readers and writers (Figure 2F) (R= 0.411143153240786, P<0.001). Further analysis using TCGA data shows a high correlation between YTHDC1 and SYNPO2L mRNA (Figure 5B). qPCR data from our patient database also reveals a high correlation (Figure 5C). Through knocking down and overexpressing YTHDC1, we observed that overexpression significantly increases SYNPO2L mRNA levels (Figure 5D), while knockdown markedly suppresses SYNPO2L mRNA expression (Figure 5E). Additionally, we aimed to further demonstrate the involvement of YTHDC1 in SYNPO2L mRNA stability. Comparing the effects of YTHDC1 knockdown and overexpression on SYNPO2L stability, we found a significant decrease in SYNPO2L stability upon YTHDC1 knockdown (Figure 5F), while overexpression notably increased stability (Figure 5G). In analyzing the stability region of the SYNPO2L gene, we identified METTL16 binding sites and designed ChIP primers by http://m6avar.renlab.org/index.html (Figure 5H). To validate the importance of this site, we mutated this site and used luciferase to verify its role (Figure 5I). We found that knocking down METTL16 reduced luciferase activity only in the wild-type, with no change upon mutating this site (Figure 5J). Overexpressing METTL16 in the non-mutated wild-type site significantly increased luciferase compared to the control (Figure 5J). In contrast, overexpression in the mutated site had no effect (Figure 5J), indicating the site's critical role in METTL16's function.

### Cancer-associated Fibroblasts (CAFs) in the Tumor Microenvironment Play a Crucial Role in SYNPO2L-Induced Distant Metastasis

**Figure 6:**
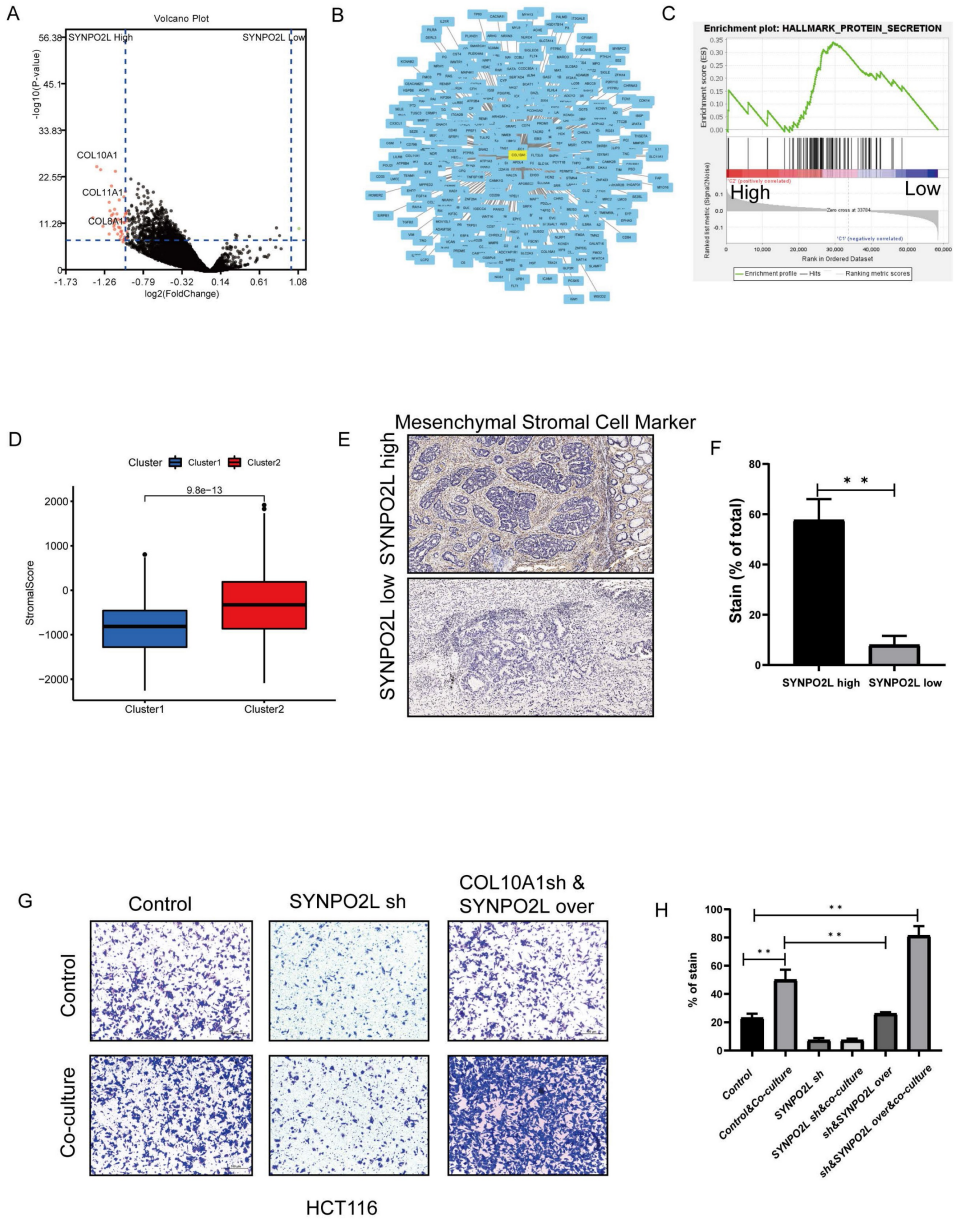
** SYNPO2L Interacts with Cancer-Associated Fibroblasts (CAFs) through Secreted Proteins.** (A) TCGA analysis of differentially expressed genes between high and low SYNPO2L expression groups, described in a volcano plot; (B) Protein-protein interaction network map; (C) TCGA enrichment analysis of high vs. low SYNPO2L expression groups; NES=1.61, NOM p-val < 0.0001. (D) TCGA analysis of stromal cell scoring in high vs. low SYNPO2L expression groups, shown in a bar graph; (E-F) Immunohistochemical analysis of CAFs markers and their representation in bar graphs; (G-H) Transwell cell migration assays to assess the impact of overexpressing/knocking down SYNPO2L on the migratory capabilities of colorectal cancer cells, displayed in bar graphs. *P < 0.05, **P < 0.01, ***P < 0.001, ****P < 0.0001. NS, No significant.

Our previous research revealed that SYNPO2L is a key factor in poor patient prognosis. However, how it works is an important topic to solve. We utilized TCGA database for WGCNA dataset analysis to explore the mechanism behind the effect of SYNPO2L. Dividing patients into two groups based on SYNPO2L expression levels, we identified a series of core genes (Figure S3A-F). Further differential analysis and integration with the STRING dataset identified COL10A1 as a core gene with differential expression and central importance (Figure 6A, B). Enrichment analysis showed a close relationship between high SYNPO2L expression and secretory proteins (Figure 6C) with COL10A1 being a significant secretory protein. Notably, COL10A1 is associated with CAFs. Calculating the CAFs Score for high SYNPO2L expression patients SYNPO2L revealed a significantly higher score compared to low-expression patients (Figure 6D), highlighting the connection between SYNPO2L and CAFs. Tissue samples from our colorectal cancer patient database also showed that high SYNPO2L expression patients have higher Mesenchymal CAFs Cell Marker levels than low expression patients (Figure 6E, F). To study SYNPO2L's role in tumor metastasis, we conducted cell migration assays. We first co-cultured wild-type tumor cells with CAFs, which shows a significantly increase of tumor cell migration compared to non-co-cultured conditions (Figure 6 G, H). However, knocking down SYNPO2L significantly reduced tumor cell migration under co-cultured conditions (Figure 6 G, H), indicating the significant impact of SYNPO2L on tumor migration. However, what is interesting is that after knockdown, followed by overexpression, the cell migration ability is the same as the control group, indicating that overexpression affects tumor cell growth but does not significantly impact tumor cell migration (Figure 6 G, H). Even more interestingly, if cells that have been knocked down are overexpressed and then co-cultured with CAF Cells, the cell migration ability is significantly enhanced compared to the control group. This suggests that SYNPO2L enhances the cell migration ability through a joint effect with CAF Cells.

### SYNPO2L Promotes Tumor Migration by Enhancing the Expression of COL10A1, which Interacts with Cancer Associated Fibroblasts (CAFs)

**Figure 7:**
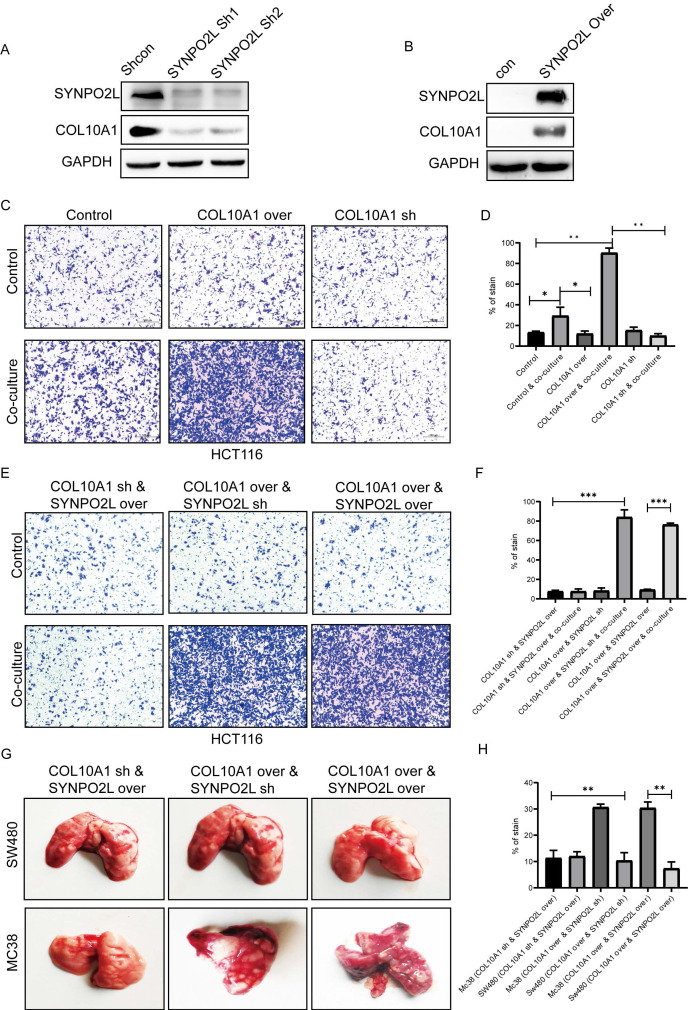
** SYNPO2L Influences Tumor Cell Secretion of COL10A1, Promoting Infiltration of Tumor-Associated Fibroblasts and Leading to Distant Tumor Metastasis.** (A-B) Western blot analysis of COL10A1 expression levels following SYNPO2L knockdown/overexpression. GAPDH used as internal control protein; (C-D) Transwell cell migration assays evaluating the impact of knocking down/overexpressing COL10A1 on colorectal cancer cell migration, shown in bar graphs; (E-F) Transwell assays assessing the impact of COL10A1 knockdown and SYNPO2L overexpression, COL10A1 overexpression and SYNPO2L knockdown, and COL10A1 and SYNPO2L overexpression on cell migration, displayed in bar graphs; (G) Tumor formation with tail vein injection in nude mice to assess the impact of COL10A1 knockdown and SYNPO2L overexpression, COL10A1 overexpression and SYNPO2L knockdown, and COL10A1 and SYNPO2L overexpression on lung metastasis, shown in a bar graph. *P < 0.05, **P < 0.01, ***P < 0.001, ****P < 0.0001.

COL10A1, known to be a crucial factor secreted by CAFs, has not been fully understood in terms of its function^15^. Our research discovered that tumor cells can also secrete COL10A1, which works in conjunction with CAFs. Whether SYNPO2L directly interacts with CAFs remained unclear, with no relevant reports. We identified COL10A1 as a target gene through which SYNPO2L exerts its effects (Figure 6B). Further experiments showed that knocking down SYNPO2L led to a decrease in COL10A1 (Figure 7A), while overexpressing SYNPO2L caused an increase in COL10A1 (Figure 7B). This suggests SYNPO2L regulates COL10A1 expression to perform its functions. Subsequent experiments revealed that COL10A1 also promotes distant metastasis only when co-cultured with CAFs (Figure 7 C, D). To determine whether SYNPO2L and COL10A1 function independently or SYNPO2L influences COL10A1, we knocked down COL10A1 and overexpressed SYNPO2L. We found this had no significant impact on tumor migration, regardless of co-culture (Figure 7 E, F). However, overexpressing COL10A1 and knocking down SYNPO2L demonstrated significant effects only in co-culture conditions (Figure 7 E, F). When both were overexpressed, the effect was similar to overexpressing COL10A1 and knocking down SYNPO2L (Figure 7 E, F). This indicates that SYNPO2L interacts with CAFs in co-culture conditions through COL10A1. To provide stronger evidence, we set up an in vivo experiment. It is challenging to simulate co-culture conditions with CAFs in human cell lines injected into the tail vein of nude mice, due to the lack of human-derived CAFs. Indeed, we observed that whether COL10A1 and SYNPO2L were overexpressed, the effect was similar to the control group in human cell line injections (Figure 7 G, H). However, when mouse-derived cell lines were injected, overexpression of COL10A1 and SYNPO2L significantly increased lung metastasis (Figure 7 G, H). This suggests that CAFs of the same species are effective in promoting tumor metastasis. Therefore, this experiment further validates the roles of COL10A1 and SYNPO2L, aligning with our in vitro findings (Figure 7 G, H).

### COL10A1 Promotes Distant Metastasis through Interaction with Cancer Associated Fibroblasts (CAFs) and the Induction of Epithelial-Mesenchymal Transition (EMT)

**Figure 8:**
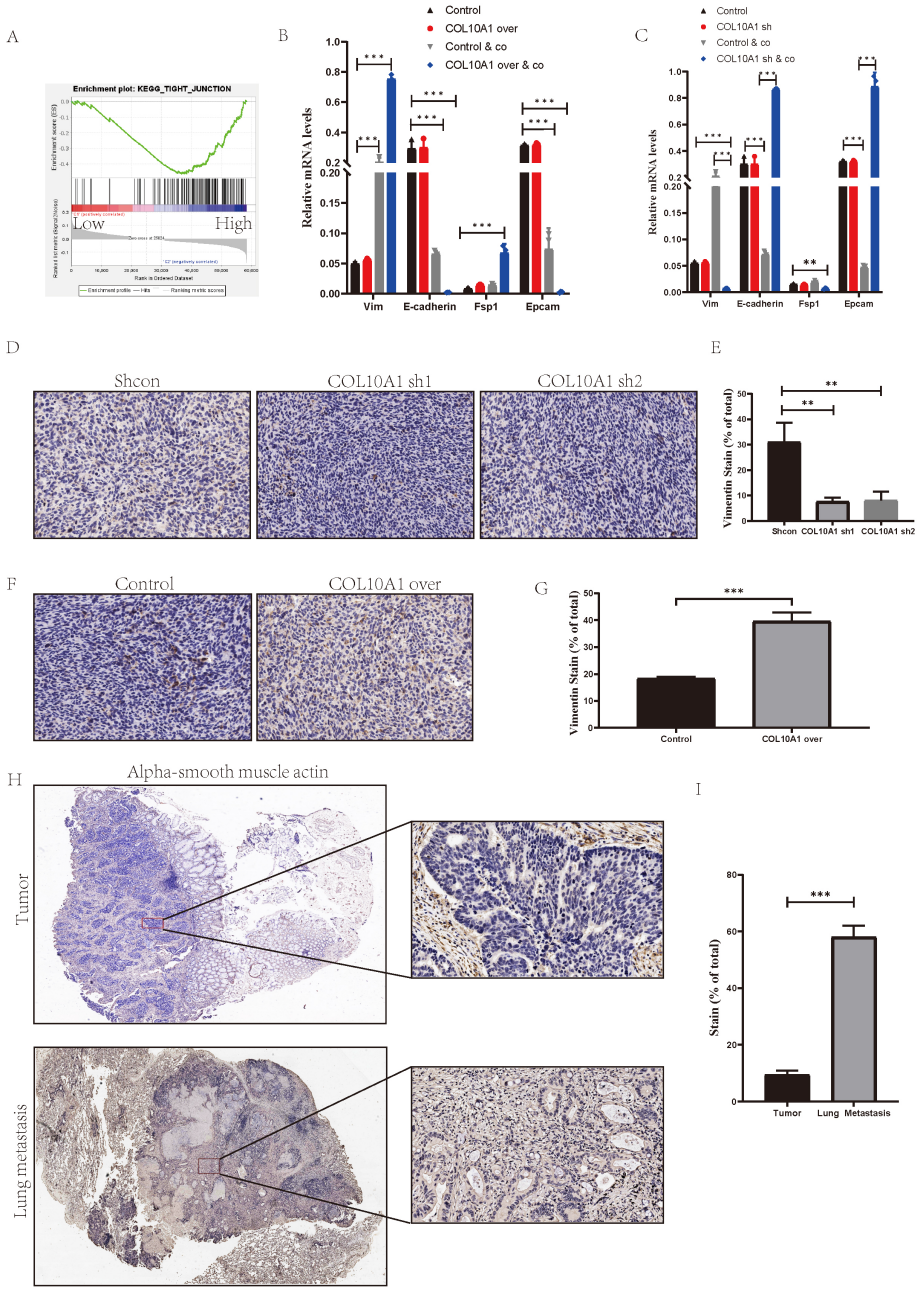
** COL10A1 Promotes Tumor-Associated Fibroblast Infiltration Leading to EMT and Metastasis in Tumor Cells.** (A) TCGA enrichment analysis of high vs. low COL10A1 expression groups; NES= 1.68, NOM p-val = 0.028. (B-C) qPCR analysis of EMT marker expression levels in colorectal cancer cells after overexpressing or knocking down COL10A1; (D-G) Subcutaneous tumor formation in MC38 cell line with COL10A1 knockdown/overexpression, followed by immunohistochemical analysis of Vimentin protein in subcutaneous grafts, shown in bar graphs; (H-I) Immunohistochemical analysis of CAFs markers in primary and lung metastatic colorectal cancer from patients, displayed in bar graphs. *P < 0.05, **P < 0.01, ***P < 0.001, ****P < 0.0001.

Our previous experiments showed that SYNPO2L, through COL10A1, interacts with CAFs to drive tumor progression and metastasis. However, the mechanism of COL10A1's interaction with CAFs was not well-documented, which further investigation into COL10A1's function. Analysis of TCGA data, based on COL10A1 expression levels, revealed that high COL10A1 expression is associated with tight junction pathways, which are closely related to mesenchymal cells (Figure 8A)^16^. However, overexpressing or knocking down COL10A1 showed no significant change in EMT markers in cells (Figure 8B, C and Figure S5A). Considering that TCGA data are from patient tumor samples, which include immune or tumor microenvironments, and our data indicating that COL10A1 requires interaction with CAFs to exhibit differential effects, we co-cultured cells with CAFs. Remarkably, co-culture induced EMT in the cells (Figure 8B, C). Overexpressing COL10A1 in this co-culture setup significantly increased mesenchymal cell markers compared to the control group (Figure 8B), indicating a transition from epithelial to mesenchymal cells. This finding aligns with our previous experimental results. When COL10A1 was knocked down in the co-culture, cells maintained an epithelial state (Figure 8C). In vivo experiments involving subcutaneous injection of MC38 cells into nude mice showed that knocking down COL10A1 increased epithelial tumor cells, while overexpression led to a notable increase in mesenchymal tumor cells (Figure 8D-G, Figure S4 A, B). More interestingly, overexpressing COL10A1 also significantly increased CAFs (Figure S5 B, C). Patient samples further revealed that, compared to primary tumors, lung metastases had a significant increase in CAFs, underscoring the crucial role of CAFs in tumor metastasis (Figure 8H, I). These findings suggest that COL10A1 can promote the chemotaxis of CAFs and, in conjunction with CAFs, facilitate the occurrence of EMT in tumors, thereby promoting tumor progression and metastasis.

## Discussion

Metastatic colorectal cancer (mCRC) remains a deadly disease with a 5-year survival rate of about 14%. While early-stage colorectal cancer can be cured through surgery or without adjuvant chemotherapy, mCRC cannot be eradicated due to the heavy burden of diffuse cancer cells, including therapy-resistant metastatic cells^17^. Thus, researching treatments for mCRC is a priority in our search for therapeutic targets. High stages of colorectal cancer are a major cause of metastasis^18^. Our study also finds that prognosis in patients with advanced colorectal cancer is significantly worse than in those with early stages. Furthermore, patients with advanced stages show strong EMT signaling pathways, a key factor in distant metastasis. But how do patients with advanced stages activate EMT and thus undergo metastasis?

With the discovery of m6A, its role in promoting tumor metastasis has been increasingly demonstrated in many studies^19^. Starting from m6A modifications, we analyzed the transcriptome of colorectal cancer patients in the TCGA database to identify genes regulated by m6A. Interestingly, we found that SYNPO2L is an important gene regulated by m6A, affecting both patient prognosis and metastasis. Our patient sample database also confirms the significant role of this gene in tumor development. However, there is limited research on this gene in tumors, and its functions are not well-documented in the literature. Interestingly, many studies have found that this gene plays a significant role in cell fibrosis or fibroblast formation^20^.

Therefore, we conducted experimental research on this gene. We found that SYNPO2L plays an important role in both the growth and metastasis of tumors. Moreover, we discovered that the regulation of this gene is determined by the newly discovered m6A writer gene, METTL16. However, whether METTL16 exerts its effects through SYNPO2L was unknown. Through experiments, we found that METTL16 cannot fully exert its intended effects in the absence of SYNPO2L. Moreover, we demonstrated that METTL16 mediates m6A modifications on SYNPO2L and completes the process with the help of YTHDC1 recognition.

Here, we have only proven the mechanism regulating SYNPO2L. However, how it functions requires further study. Continuing our analysis of the TCGA colorectal cancer patient transcriptome, we found that SYNPO2L can promote the expression of certain secretory proteins and is an important gene affecting protein secretion. Interestingly, we discovered that COL10A1 is a core gene regulated by SYNPO2L. This gene is highly related to the function of fibroblasts. As mentioned earlier, many studies have found that SYNPO2L plays a significant role in fibrosis in normal tissues or in fibroblasts. Moreover, we found that in patients with high expression of SYNPO2L, its expression is significantly related to fibroblasts. Thus, we strongly suspect that SYNPO2L regulates tumor-associated fibroblast infiltration by controlling COL10A1, thereby playing its role.

Therefore, we conducted several experiments and found that SYNPO2L regulates the secretion of COL10A1 by tumor cells, thus guiding the occurrence of tumor metastasis. Collagen type X (COL10) is a homotrimeric non-fibrillar collagen found in the human body and belongs to the collagen protein family. The alpha 1 chain of collagen type X (COL10A1) is a specific cleavage fragment of collagen type X^21^. In recent years, research on the role of COL10A1 in tumors has increased. High expression of COL10A1 protein in cancer tissues has been confirmed to be associated with tumor angiogenesis in different types of cancer^22^.

We initially discovered that patients with advanced stages are more likely to undergo distant metastasis, leading to poor prognosis. Metastasis in these patients is mainly due to EMT. Analyzing the transcriptome data of TCGA colorectal cancer patients, we found that patients with high expression of COL10A1 have an open tight junction signaling pathway, which is closely related to EMT. Furthermore, our subsequent experiments also found that tumors secrete COL10A1, which, in conjunction with tumor-associated fibroblasts, guides epithelial tumor cells towards mesenchymal tumor cells, thus mediating tumor cell metastasis.

Although our research has shown that METTL16 modifies SYNPO2L at the mRNA level through m6A, enabling SYNPO2L to function more effectively, and SYNPO2L promotes the secretion of COL10A1 and the infiltration of tumor-associated fibroblasts, thereby facilitating EMT in tumor cells and making them more prone to distant metastasis. However, we are not yet clear on how SYNPO2L promotes the secretion of COL10A1. More research is needed to support this in the future (Figure S6).

### Data Availability Statement

The datasets presented in this study can be found in online repositories. The names of the repository/repositories and accession number(s) can be found in the article/ supplementary material.

## Supplementary Material

Supplementary figures.

## Figures and Tables

**Table 1:** Antibody Information

Antibody	Company	Catalog number
METTL16	Abcam	ab313743
E-cadherin	Proteintech	20874-1-AP
SYNPO2L	Proteintech	21480-1-AP
Vimentin	Abcam	ab92547
S100A4	CST (Cell Signaling Technology)	#13018
α-Smooth Muscle Actin	CST (Cell Signaling Technology)	#19245
GAPDH	ABCAM	ab8245
