# Supplementary material for: METTL16 Promotes Stability of SYNPO2L mRNA and leading to Cancer Cell Lung Metastasis by Secretion of COL10A1 and attract the Cancer-Associated Fibroblasts
Source: Int J Biol Sci. 2024 Aug 1;20(11):4128–45. doi: 10.7150/ijbs.95375 (PMC11379079; doi:10.7150/ijbs.95375)
Supplement: Supplementary file 1 — Supplementary figures. [file ijbsv20p4128s1.pdf]

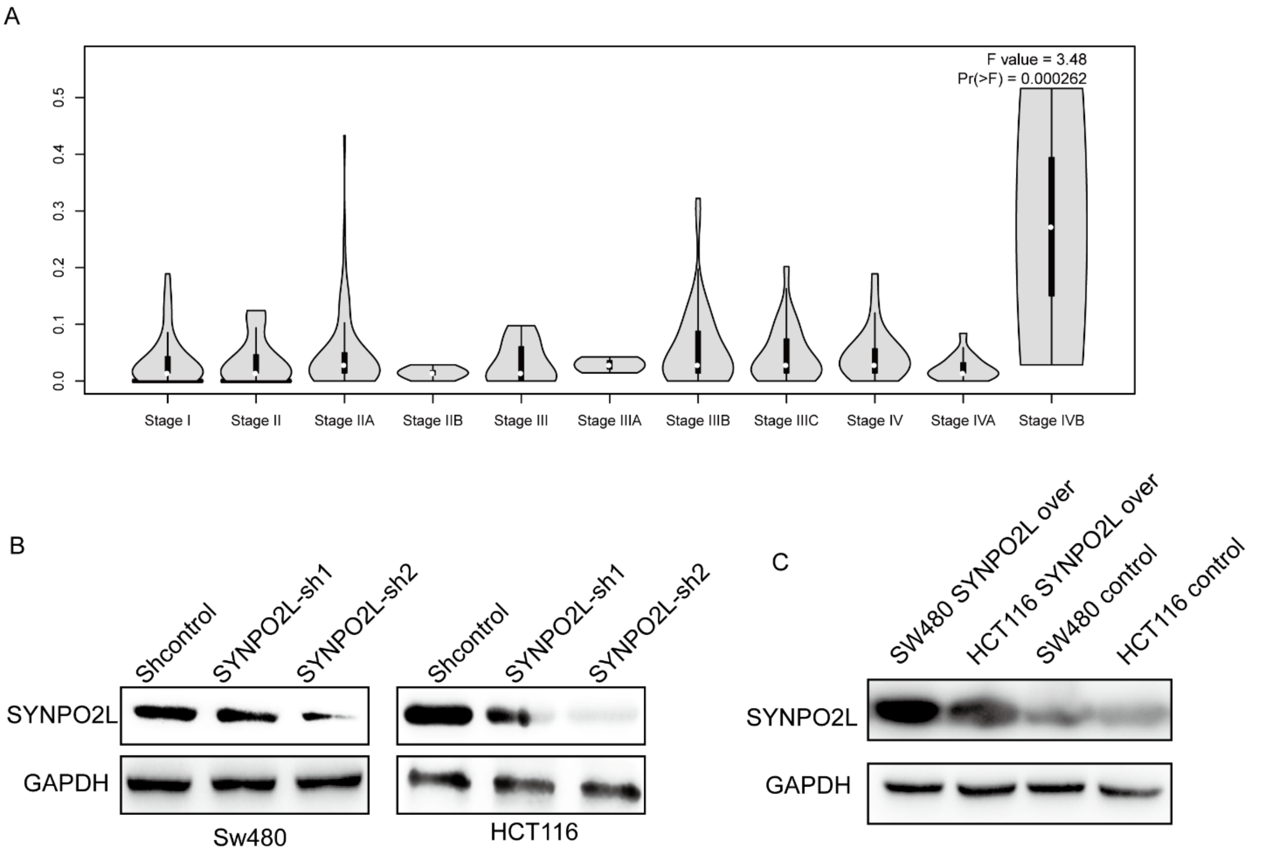

Figure S1. SYNPO2L significant correlation with the late stages of patients in TCGA (A). Western Blot Validation of SYNPO2L Knockdown and Overexpression (B, C). (B) Western blot analysis of SYNPO2L silencing efficiency in SW480 and HCT116 cell lines. GAPDH used as an internal control protein; (C) Western blot analysis of SYNPO2L overexpression efficiency in SW480 and HCT116 cell lines. GAPDH used as an internal control protein.

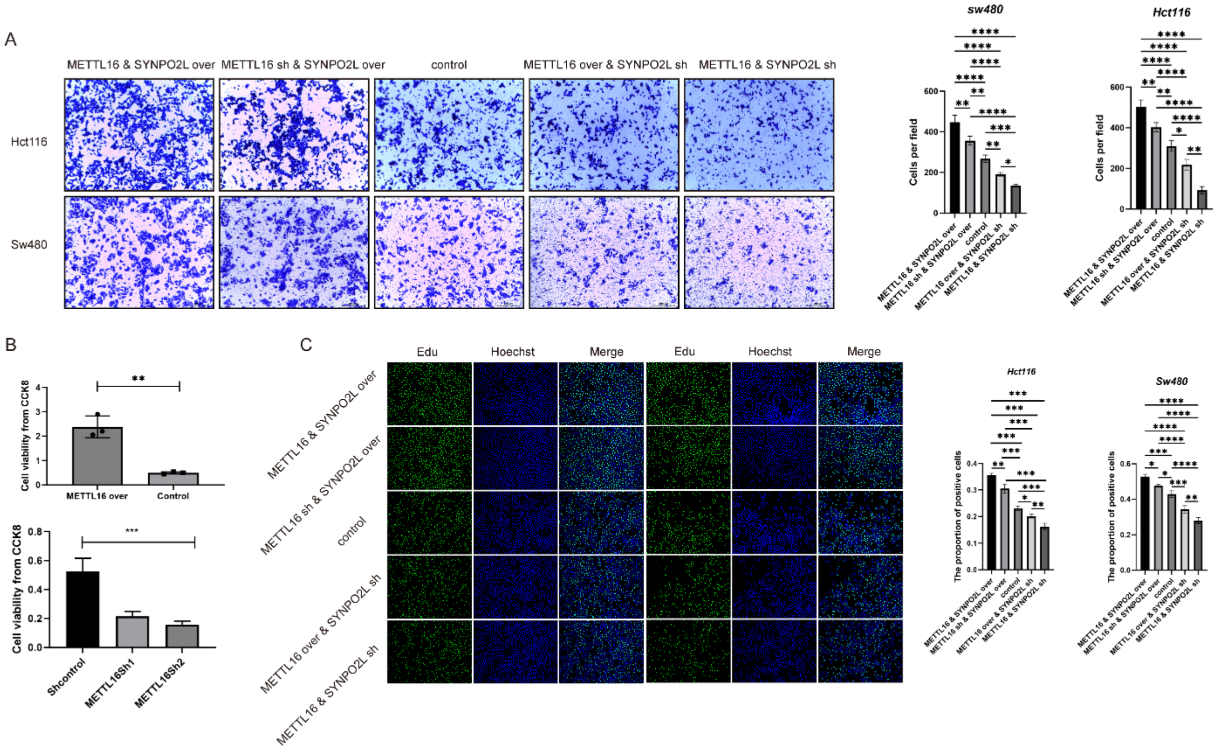

Figure S2. METTL16 Affects cells viability and SYNPO2L Function. (A) Transwell migration experiment and statistical bar graph for simultaneous knockdown of METTL16 and SYNPO2L, overexpression of METTL16 and SYNPO2L, knockdown of METTL16 with overexpression of SYNPO2L, and overexpression of METTL16 with knockdown of SYNPO2L. (B) CCK8 experiments and statistical bar graphs for overexpression and knockdown of Metll16. (C) EDU experiment and statistical bar graph for simultaneous knockdown of METTL16 and SYNPO2L, overexpression of METTL16 and SYNPO2L, knockdown of METTL16 with overexpression of SYNPO2L, and overexpression of METTL16 with knockdown of SYNPO2L. *$P < 0.05$, **$P < 0.01$, ***$P < 0.001$, ****$P < 0.0001$.

A

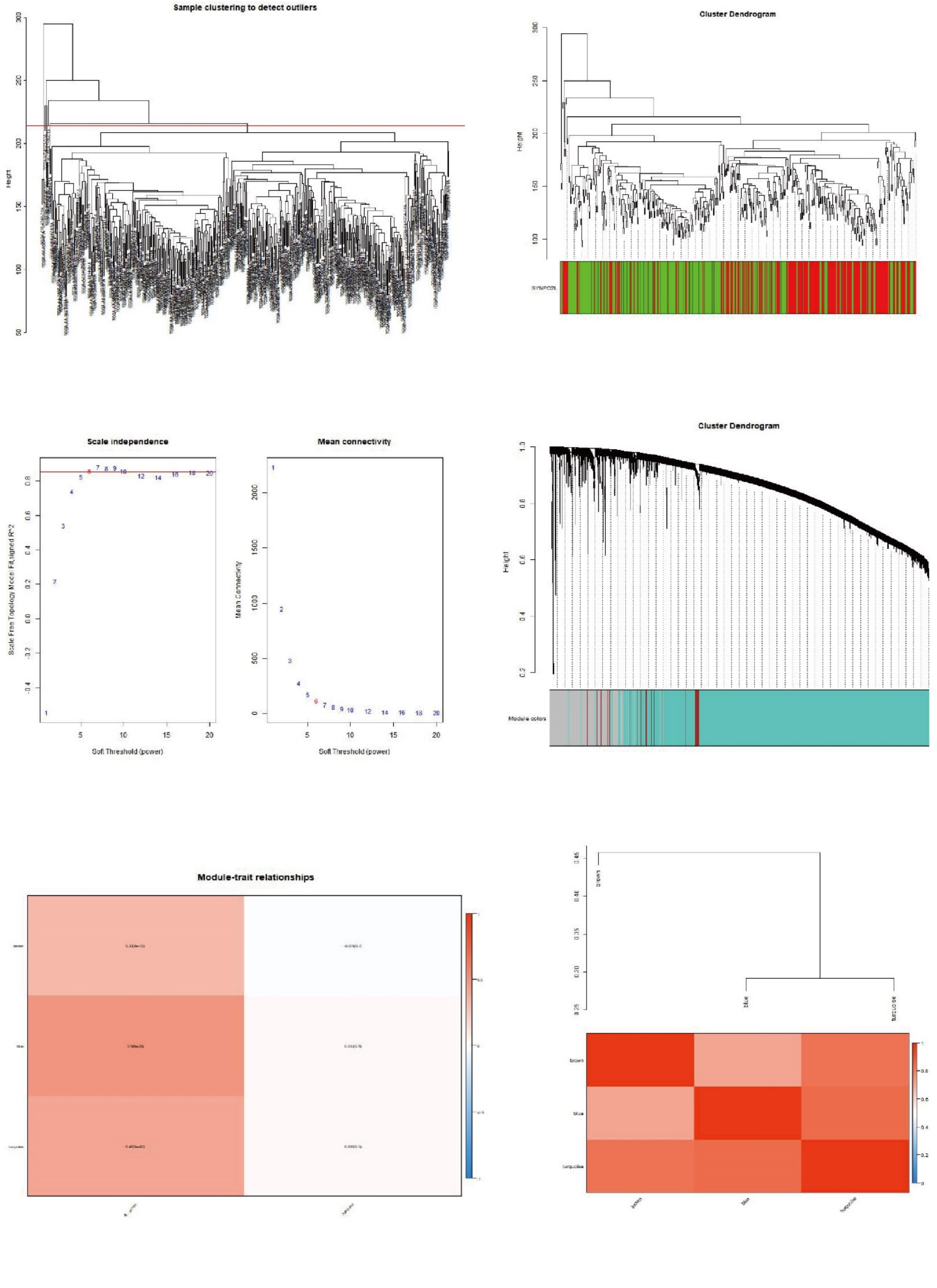

Sample clustering to detect outliers
Height
Cluster Dendrogram
Height
SYNPO2L
Scale independence
Scale Free Topology Model Fit,signed R^2
Soft Threshold (power)
Mean connectivity
Mean Connectivity
Soft Threshold (power)
Cluster Dendrogram
Height
Module colors
Module-trait relationships
brown
blue
turquoise

Figure S3. COL10A1 is a Core Gene in Patients with High SYNPO2L Expression. (A) Dendrogram of module characteristic genes; (B) Hierarchical clustering tree, where color intensity correlates with expression levels; (C) Selection of soft-thresholding power β=6 as the minimal β for a scale-free network topology；(D) Hierarchical clustering dendrogram of module genes, with each branch representing a gene and each color representing a co-expression module; (E) Heatmap showing the correlation of each module's characteristic genes with high and low SYNPO2L expression groups, with deep orange indicating a positive correlation with the corresponding group; (F) Heatmap displaying the correlation analysis for each module.

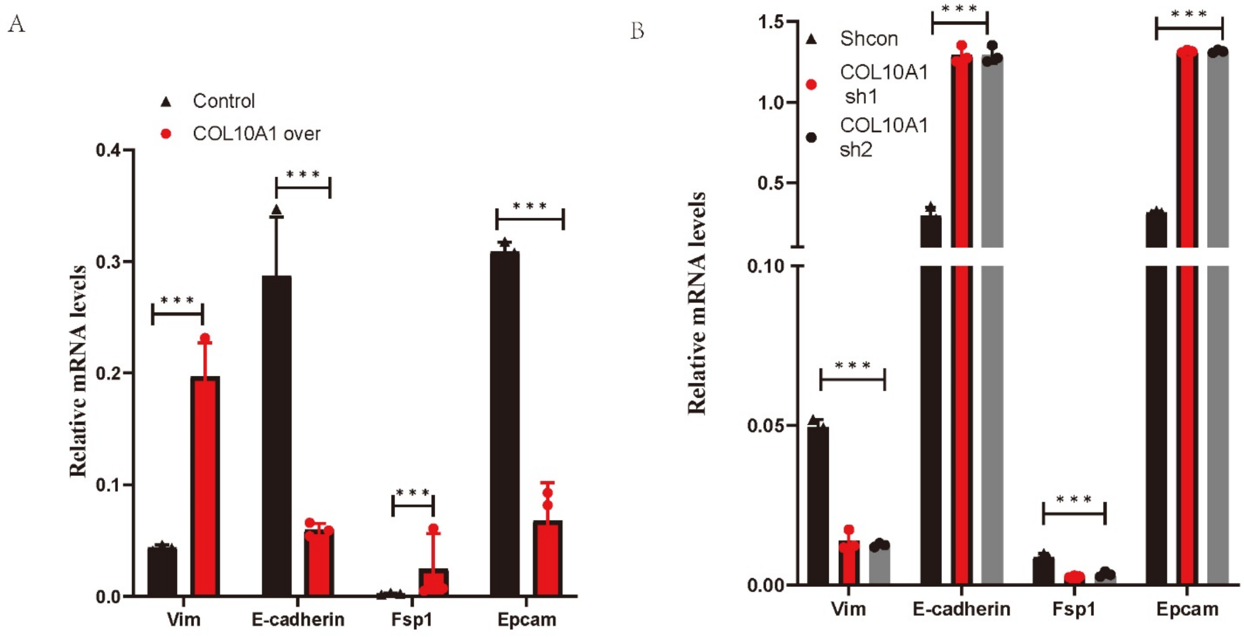

Figure S4. COL10A1 Promotes EMT Through Tumor-Associated Fibroblasts. (A-B) qPCR analysis of CAFs marker expression levels in MC38 cells after overexpression or knockdown of COL10A1. *$P < 0.05$, **$P < 0.01$, ***$P < 0.001$, ****$P < 0.0001$.

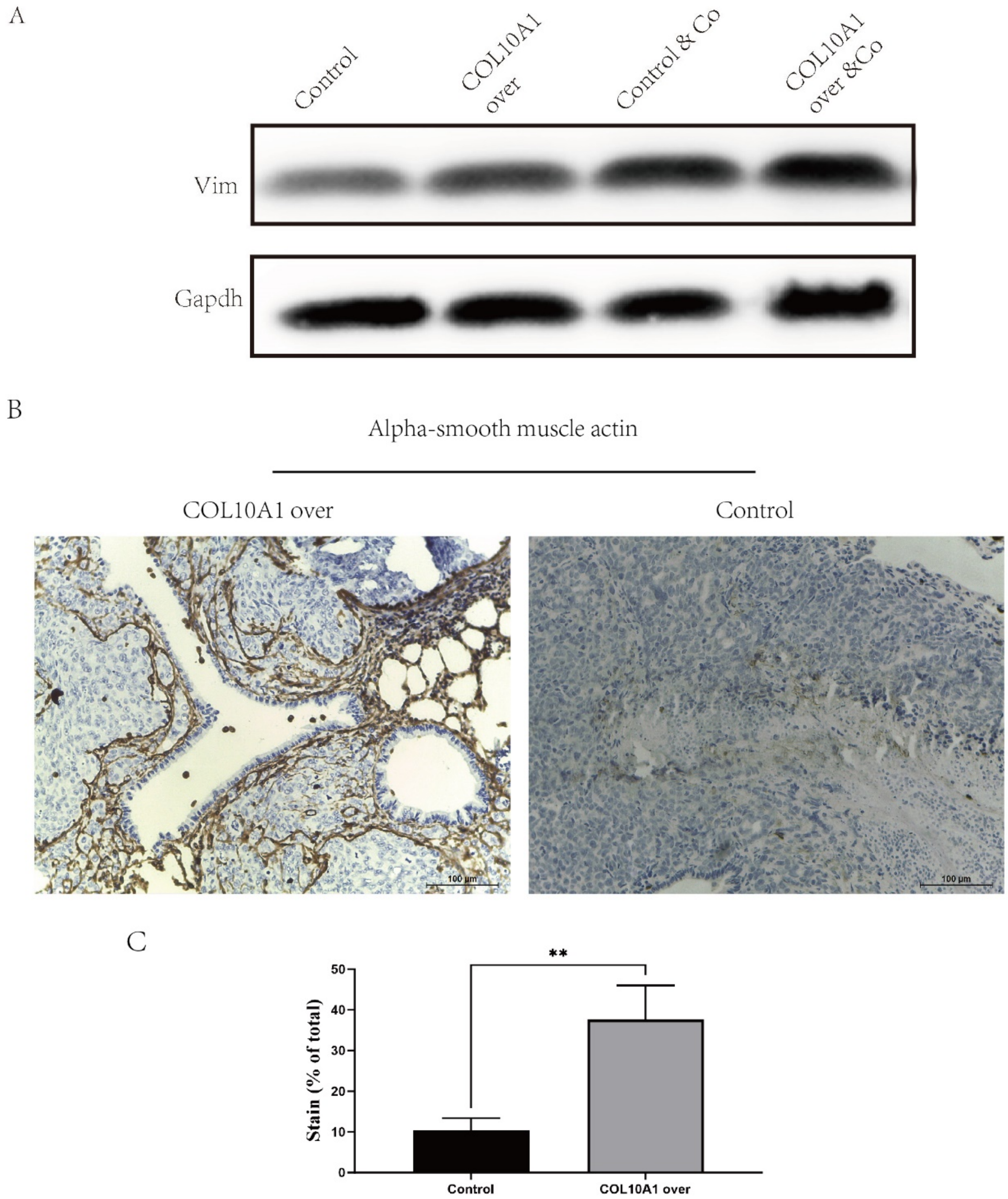

Figure S5. Overexpression of COL10A1 Significantly Promotes Infiltration of Tumor-Associated Fibroblasts. (A) WB analysis of EMT marker VIM expression levels in colorectal cancer cells after overexpressing COL10A1 with co-culture. (B-C) Subcutaneous tumor formation in the MC38 cell line following COL10A1 overexpression, with immunohistochemical analysis of CAFs markers in subcutaneous grafts, presented in bar graphs. *$P < 0.05$, **$P < 0.01$, ***$P < 0.001$, ****$P < 0.0001$.
